# Exercise and Progressive Supranuclear Palsy: the need for explicit exercise reporting

**DOI:** 10.1186/s12883-019-1539-4

**Published:** 2019-11-29

**Authors:** Susan C. Slade, Martin Underwood, Jennifer L. McGinley, Meg E. Morris

**Affiliations:** 1 La Trobe Centre for Sport and Exercise Medicine Research, School Allied Health, Human Services and Sport, College Science, Health & Engineering, La Trobe University, Kingsbury Drive, Bundoora, 3086 Australia; 2 Warwick Clinical Trials Unit, Division of Health Sciences, Warwick Medical School, University of Warwick, Coventry, UK; 3 Physiotherapy, The University of Melbourne, Level 7, Alan Gilbert Building, Barry Street, Parkville, Australia; 4 Healthscope, North Eastern Rehabilitation Centre, Ivanhoe, Australia

**Keywords:** Progressive Supranuclear palsy, Atypical parkinsonism, Exercise, Rehabilitation

## Abstract

**Background:**

Progressive Supranuclear Palsy (PSP) is the most frequent form of atypical Parkinsonism. Although there is preliminary evidence for the benefits of gait rehabilitation, balance training and oculomotor exercises in PSP, the quality of reporting of exercise therapies appears mixed. The current investigation aims to evaluate the comprehensiveness of reporting of exercise and physical activity interventions in the PSP literature.

**Methods:**

Two independent reviewers used the Consensus on Exercise Reporting Template (CERT) to extract all exercise intervention data from 11 studies included in a systematic review. CERT items covered: ‘what’ (materials), ‘who’ (instructor qualifications), ‘how’ (delivery), ‘where’ (location), ‘when’, ‘how much’ (dosage), ‘tailoring’ (what, how), and ‘how well’ (fidelity) exercise delivery complied with the protocol. Each exercise item was scored ‘1’ (adequately reported) or ‘0’ (not adequately reported or unclear). The CERT score was calculated, as well as the percentage of studies that reported each CERT item.

**Results:**

The CERT scores ranged from 3 to 12 out of 19. No PSP studies adequately described exercise elements that would allow exact replication of the interventions. Well-described items included exercise equipment, exercise settings, exercise therapy scheduling, frequency and duration. Poorly described items included decision rules for exercise progression, instructor qualifications, exercise adherence, motivation strategies, safety and adverse events associated with exercise therapies.

**Discussion:**

The results revealed variability in the reporting of physical therapies for people living with PSP. Future exercise trials need to more comprehensively describe equipment, instructor qualifications, exercise and physical activity type, dosage, setting, individual tailoring of exercises, supervision, adherence, motivation strategies, progression decisions, safety and adverse events.

**Conclusion:**

Although beneficial for people living with PSP, exercise and physical therapy interventions have been inadequately reported. It is recommended that evidence-based reporting templates be utilised to comprehensively document therapeutic exercise design, delivery and evaluation.

## Background

Progressive Supranuclear Palsy (PSP) is comparatively rare. Nevertheless, it is the most frequent form of atypical Parkinsonism [1–3]. The prevalence estimates range from 5 to 6 per 100,000 [4] to 18 per 100,000 [5]. Although difficult to diagnose the International Parkinson and Movement Disorders Society (MDS-PSP) clinical criteria have provided expert guidance [6]. Given the rapid decline in movement control, balance, and oculo-motor control in people with PSP, exercise and physical activities may be important to health and wellbeing [7, 8]. People with PSP also have an increased risk of falls [7, 8]. Strength training, gait rehabilitation, falls education and other forms of exercise and physical activity [7, 8], alongside optimal medical management, are argued to improve quality of life for people with this debilitating condition [7, 8].

Exercise therapy can be helpful for people with idiopathic Parkinsonism [9, 10]. Physiotherapy that includes treadmill gait training, cueing, dance and martial arts has benefits for gait, balance and quality of life in some individuals [11]. In addition, progressive resistance strength training is beneficial for gait, falls prevention and community ambulation [12–14]. High-intensity interval treadmill training is safe, feasible and beneficial for motor symptoms such as gait and balance in the early stages of idiopathic Parkinson’s disease (PD) [15]. Falls prevention interventions for PD, including progressive resistive strength training, movement strategies and education, have shown reduced falls rates when delivered in outpatient settings [9]. In clinical settings people living with PSP often receive an idiopathic PD exercise and movement rehabilitation program because initial symptoms or difficulty with function and activity limitations can appear similar [16, 17]. Whether or not this is appropriate is open to question.

Exercises and structured physical therapies are also thought to be beneficial for individuals living with PSP [16–19], yet the evidence is based on a small number of underpowered and heterogeneous studies [20]. In addition, these studies have not used a consistent core set of outcome measures for disability, falls, movement disorders, wellbeing and quality of life [20]. Some balance, gaze and gait variables have been measured although they have not been reported in a consistent manner [20]. There is preliminary evidence in small observational studies for the benefits of oculomotor exercises for balance and walking speed in PSP [21, 22]. Gait training and cueing have demonstrated benefits for balance, reduced falls and quality of life [23–26]. For findings to be understood, and to inform components for future PSP trials, it is important to report how exercises are designed, delivered and evaluated.

Best practice for exercise, rehabilitation and physical activity programs is to use an evidence-based reporting template to detail the exercise type, content, setting, instructor qualifications and skills, exercise equipment, dosage, progression decision rules, and motivation and adherence strategies to be used by patients and therapists [27–30]. For exercise programs to be safely and effectively translated into clinical practice, several important intervention elements need to be described [28, 29]. The Consensus on Exercise Reporting Template (CERT) provides a framework for reporting these elements [28–30]. The template affords a standardised method for exercise data extraction across a range of study designs. It assists readers, peer-reviewers and editors with the appraisal of exercise reporting quality and reporting of the content of exercise programs [28]. The template can also guide exercise protocol development and the structuring of recommendations for clinical implementation [28].

The aim of the current investigation is to evaluate the comprehensiveness of reporting of exercise and physical activity interventions reported in the PSP literature.

## Materials and methods

### Systematic review

As a first step in this analysis we conducted a systematic review of exercise and physical activity interventions for people with PSP. This has been reported in detail elsewhere [20] and is summarised as follows. The protocol was pre-registered on PROSPERO (CRD42018103845) (https://www.crd.york.ac.uk/prospero), methods followed Cochrane guidelines [31] and reporting followed Preferred Reporting Items for Systematic review and Meta-Analysis (PRISMA) guidance [32, 33]. Two reviewers independently conducted all of the review stages and agreement was reached by discussion and research team consensus. English language, peer-reviewed published studies of any design, and conducted in any setting, were eligible for inclusion. Nine electronic databases were searched until August 18, 2019 using terms such as exercise, physical activity, rehabilitation, and synonyms for PSP. Reference lists were searched and content experts were consulted.

### CERT data extraction and analysis of exercise interventions

Two reviewers (SCS and MEM) independently extracted all exercise intervention component data from the included studies by using the 16-item CERT checklist [27]. CERT items span the following domains: ‘what’ (materials), ‘who’ (instructor qualifications), ‘how’ (delivery), ‘where’ (location), ‘when’, ‘how much’ (dosage), ‘tailoring’ (what, how), and ‘how well’ (fidelity). The CERT Explanation and Elaboration statement [29] provided further guidance. If information was missing, one reviewer (SCS) searched for, and retrieved, any published protocols, online appendices and supplementary data for additional data extraction. Each item was scored ‘1’ (adequately reported) or ‘0’ (not adequately reported or unclear), and a score out of a total of 19 was calculated. The independently extracted data were examined for consistency and any discrepancies were resolved through discussion between the reviewers.

Intervention content was summarised in table format under headings such as study design and sample size, setting, type of exercise and mode of delivery, program duration and dosage. The quality and comprehensiveness of reporting were analysed and presented using the example of McGregor et al. [34]. The CERT score (with a possible total of 19) and the number (and percentage) of studies that reported each CERT item were calculated, summarised in narrative form and reported graphically.

## Results

### Systematic review

The results of the systematic review have been reported in detail elsewhere [20] and are summarised as follows. There were 11 included studies, with an overall total of 99 participants [21–26, 35–39]. All studies were assessed as having a moderate to high risk of bias. Study designs included randomised/quasi-randomised controlled trials, quasi-experimental studies, cohort and case studies. The intervention characteristics are described in Table 1. Intervention effects were typically small to moderate or not statistically significant. There was preliminary evidence that treadmill training with harness support, auditory-cued motor training and robot-assisted gait rehabilitation might be helpful for some people in the early stages of PSP [20]. The systematic review found insufficient evidence of benefits of movement rehabilitation in the advanced stages of PSP [20]. It also remains unclear whether therapeutic exercises shown to be effective for people living with idiopathic Parkinson’s disease benefits those with PSP [20].

**Table 1:** Intervention characteristics

Study	Sample size	Study design	Setting	Exercise	Modality	Program duration	Dosage
Clerici 2017, Italy [23]	24	RCT	Rehabilitation hospital	Gait training - aerobic, intensive, motor-cognitive, goal based exercise	Treadmill versus Lokomat robotic treadmill	4 weeks	4 daily 1-h sessions, 5/week
Di Pancrazio 2013, Italy [39]	10	Cohort study	Rehabilitation hospital	Weight relieving harness for walking (SPAD) plus vibration sensory stimulation (VISS)	Treadmill	8 weeks	3/week
Irons 2015, USA [36]	1	Case study	Outpatient	Weight relieving harness for walking on a treadmill type elliptical walker/trainer	Elliptical cross trainer	8 weeks	3/week for a total of 24 sessions
Nicolai 2010, Germany [24]	8	Cohort study	Outpatient	Dynamic exercises from 6 posture & balance categories with increasing difficulty & complexity	(1) sitting (2) standing (3) transfers (4) sway (5) reaching or stepping one direction (6) multi direction stepping with added limb movement	6 weeks	45 min sessions, 3/week
Sale 2014, Italy [25]	5	Cohort study	Outpatient	Program of robot-assisted walking sessions for 20 - 45 min, 5 times a week for 4 weeks.	Walkway force platform	4 weeks	20-45 min, 5/week
Seamon 2017, USA [37]	1	Case study	In-home	Virtual exer-gaming “YourShape” and mini games	Xbox Kinect	6 weeks	12 1-h sessions
Suteerawattananon 2002, USA [35]	1	Case study	Research laboratory	Body weight supported unloaded gait	Pacer treadmill	8 weeks	1.5 h sessions, 3/week
Wallace 2013, USA [38]	1	Case study	In-home	Weighted vest, with motion-capture technology, to improve movement and posture	Weighted vest on or off	Not reported	Not reported
Wittwer 2018, Australia	5	Case series	In-home	Gait training program and rhythmic auditory cues (RACs)	Cued exercises in sitting, standing, walking	4 weeks	30–60 min, 2/week
Zampieri 2008, Italy [21]	19	Quasi RCT	Research laboratory	Exercises included tandem stance practice with eyes open and closed, turning 360° while marching in place; sit-to-stand and stand-to-sit practice on a chair. Treatment group received eye movement & visual awareness training	Balance vs balance + eye movement	4 weeks	1 h, 3/week
Zampieri 2009, Italy [22]	19	Quasi RCT	Research laboratory	As above	Balance vs balance + eye movement	4 weeks	1 h, 3/week

### CERT data extraction and data analysis

**Fig. 1:**
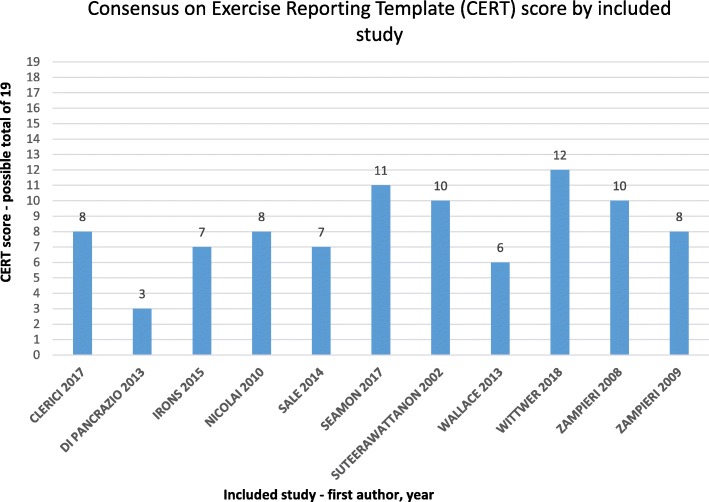
Consensus on Exercise Reporting Template applied to included studies

**Fig. 2:**
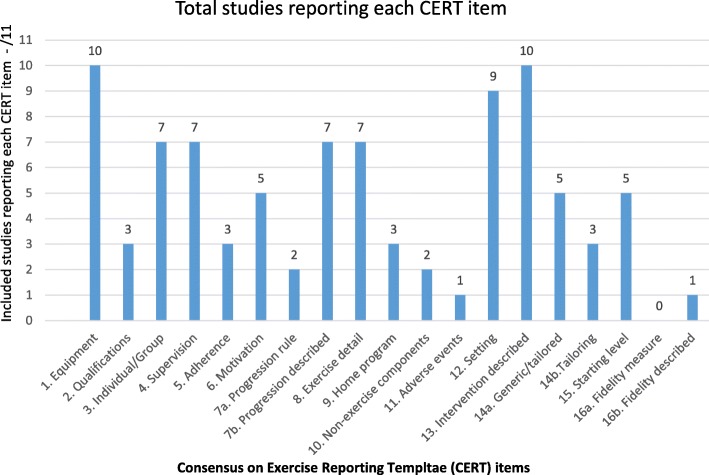
Reporting quality of exercise interventions as assessed with the Consensus on Exercise Reporting Template (CERT)

The CERT items reported for each study are summarised in Table 2. The CERT scores ranged from three to 12 out of a possible 19 points (Fig. 1). None of the included studies adequately described the minimum number of elements considered by the CERT panel to enable replication; either, clinically, or in research (Fig. 2). The authors of one study provided additional online data with an intervention description [23]. We did not find any published protocol papers.

**Table 2:** Study reporting of each CERT item

CERT item	Clerici 2017	Di Pancrazio 2013	Irons 2015	Nicolai 2010	Sale 2014	Seamon 2017	Suteerawattanon 2002	Wallace 2013	Wittwer 2019	Zampieri 2008	Zampieri 2009
1. Equipment	✓	✓	✓	✓	✓	✓	✓	X	✓	✓	✓
2. Qualifications	✓	X	X	X	X	X	X	X	✓	✓	X
3. Individual/Group	X	X	✓	X	X	✓	✓	✓	✓	✓	✓
4. Supervision	✓	X	X	X	X	✓	✓	✓	✓	✓	✓
5. Adherence	X	X	X	X	X	X	✓	✓	✓	X	X
6. Motivation	✓	X	X	X	X	✓	✓	X	✓	✓	X
7a. Progression rule	X	X	✓	X	X	✓	X	X	X	X	X
7b. Progression described	✓	X	✓	✓	✓	✓	✓	X	X	✓	X
8. Exercise detail	X	X	X	✓	✓	✓	✓	X	✓	✓	✓
9. Home program	X	X	X	X	X	✓	X	✓	✓	X	X
10. Non-exercise components	✓	X	X	X	X	X	X	X	✓	X	X
11. Adverse events	X	X	X	✓	X	X	X	X	X	X	X
12. Setting	X	✓	✓	✓	✓	✓	X	✓	✓	✓	✓
13. Intervention described	✓	✓	X	✓	✓	✓	✓	✓	✓	✓	✓
14a. Generic/tailored	X	X	X	✓	X	✓	✓	X	X	✓	✓
14b.Tailoring method	X	X	✓	✓	X	X	X	X	X	X	✓
15. Starting level	✓	X	✓	X	✓	X	✓	X	✓	X	X
16a. Fidelity measure	X	X	X	X	X	X	X	X	X	X	X
16b. Fidelity described	X	X	X	X	✓	X	X	X	X	X	X

✓ reported CERT item**,** X did not report CERT item

The well-described items included the equipment (such as treadmills, weighted vests and virtual gaming), setting (in home or at movement disorders clinics and hospital outpatient departments). The session durations ranged from 20 to 60 min, the intervention frequency ranged from two to five sessions per week and the intervention duration ranged from four to 8 weeks. A decision rule by which to determine the starting level and exercise progression, occurrence and type of exercise-related adverse events and fidelity of the actual intervention to the planned intervention were rarely described. The comprehensiveness of reporting for each of the CERT items is summarised under abbreviated headings in the following text.

#### Item 1: description of exercise equipment

The equipment was described in detail in 10/11 (91%) of studies and included brands and models. For example, photographs of bodyweight-supported treadmills [35], elliptical trainers [36] and audio biofeedback [24] provided clarity.

#### Item 2: investigator qualifications

The specific medical or allied health discipline was reported in 3/11 (27%) of studies [21, 23, 26]. The level of post-graduate education, years of experience in movement disorders rehabilitation and the use of intervention training programs and manuals were not described in any report.

#### Item 3: individual or group exercise

While often not specifically described, it was evident from the single case study design [24, 35–38] and case series investigations [26] that some exercise interventions were performed individually (7/11 (64%)). The RCTs were inconsistent with reported individual or group administration [21, 22] and unreported mode of delivery [23].

#### Item 4: supervision

The type of supervision was reported in 7/11 (64%) studies however this was of limited detail. Nicolai 2010 reported 1:1 supervision and Wittwer et al. reported individual home visits by a physiotherapist [24, 26]. Complex equipment [23, 25, 35, 36, 39] was assumed by the reviewers to require supervision at a clinic. The home-based programs did not report the levels of supervision required by a therapist or care-giver [26, 37, 38].

#### Item 5: exercise adherence

Measurement of adherence to the exercise or physical therapy program was reported in only 3/11 (27%) studies [26, 35, 38]. The way in which adherence was measured, such as exercise diaries, was not reported either a priori in the methods or by confirmation in the results.

#### Item 6: motivation strategies

Motivation strategies, such as goal setting, positive reinforcement, attendance at support groups or tele-coaching were not explicitly reported in any publications. However, visual and auditory cues were reported in 5/11 (45%) studies [21, 23, 26, 35, 37]. A virtual reality gaming environment was selected as a specific motivational tool [37]. Auditory and visual cueing could be considered as a motivational strategy or a motor control technique. Nevertheless, a more comprehensive description of the cues would assist with replication.

#### Item 7a: exercise progression

A priori decision rules for program progression were not well reported (2/11, 18% of studies) [36, 37]. That the interventions were progressed was reported in 7/11 (64%) studies [14, 23–25, 35–37]. Progression was generally informed by subjective assessment rather than by a validated measurement scale.

#### Item 8: detailed exercise description

The exercises or activities were reported in detail in 7/11 (64%) of the studies [21, 22, 24–26, 35, 37]. In the existing literature, several trials included treadmill walking and needed little extra detail. However, when exercises included gait, motor performance, balance training or functional activities there was generally a lack of detailed description.

#### Item 9: additional home program

Additional home-based or community-based activities or therapies were reported in 3/11 (27%) of the studies [26, 37, 38]. However explicit detail, that could explain any confounding effects, were not provided.

#### Item 10: non-exercise components

Non-exercise components such as environmental modifications, education about the condition and self-management such as speech and swallowing strategies were reported in only 2/11 (18%) of the studies [23, 26].

#### Item 11: risks and adverse events

The presence of exercise-related adverse events was reported in only 1/11 (9%) study [25]. The absence of adverse events reporting is of concern because people living with a diagnosis of PSP can have falls risks, comorbidities, pain, immobility and cognitive impairment.

#### Item 12: environment and setting

The exercise setting was well described in 9/11 (82%) of the studies. These included home-based interventions [26, 37, 38], movement disorders clinics and hospital outpatient departments [23–25, 36, 39] and research laboratories [21, 22, 35].

#### Item 13: intervention details

The frequency and duration of the interventions was well described in 10/11 (91%) studies. Most were of 4 to 8 weeks duration and delivered two to three times per week. One study was of unreported duration [38].

#### Item 14: generic exercises or personalised to individual needs

It was unclear, even with the small sample sizes such as single case studies, that there was tailoring to meet individual needs and capabilities in all of the studies. This was explicitly stated in 5/11 (45%) studies [21, 22, 24, 35, 37]. The method for tailoring to the individual was reported in 3/11 (27%) studies [22, 24, 36].

#### Item 15: intervention starting level

Testing of pre-study participant physical capability, such as gait speed and quality, was reported in 5/11 (45%) studies [23, 25, 26, 35, 36]. The stage of disease progression and weight-bearing and balance capacity were not always documented.

#### Item 16: intervention delivered as planned

Deviations from the planned intervention can occur in practice and may influence the observed effect. None of the studies reported how investigator adherence to the research protocol was measured 0/11 (0%). One study (9%) provided an online a priori protocol and reported that the intervention was delivered as planned [23].

## Discussion

This analysis highlights the need for explicit reporting of therapeutic exercises and structured physical activities in PSP studies. This would enable more rigorous evaluation of the outcomes of exercises and physical activities for people with neurological conditions such as atypical Parkinsonism. People with PSP, as with those who have idiopathic PD, need access to evidence-based therapies that are personalised to their individual needs. This in turn relies upon clinicians having access to clearly reported findings that describe all key dimensions of exercise and physical therapy.

The data extraction and analysis identified some well-described exercise program elements in the existing PSP literature. These included the exercise equipment, the setting and the intervention frequencies and durations. The less well described items were the investigator qualifications and training, a priori methods for intervention dosage and progression, tailoring exercises to individual needs, motivation and adherence strategies and non-exercise components, such as cueing, patient education and self-management. The poorly reported items were exercise-related adverse events and the fidelity of implementation of the planned exercise protocols.

The less well-described items may be elaborated by the following additional information. A statement of the instructor expertise and any intervention-specific training would indicate whether additional expertise is required. Exercise progression can be guided by measures such as the Borg Rating of Perceived Exertion Scale [40]. Adherence to exercises can be recorded in exercise diaries as attendance or actual performance i.e. number of exercise repetitions. Motivation strategies to encourage persistence with exercises can include interventions such as goal setting with input from the patient and carers, positive feedback about performance by using, for example, quantifiable data from outcome measures such as function, quality of life, gait quality and speed [41, 42], motivational interviewing [43] and ‘patient contracts’ such as written agreements about perceived ability, goals and preferred outcomes [44].

The types of interventions included in our PSP systematic review often used complex equipment at a movement disorders clinic or hospital outpatient department. Very few described home based programs or easily accessible equipment that could be used in the community. The benefits of physical activity and strength training are well established for general health and well-being in older adults [45, 46]. It was surprising that basic forms of exercise such as progressive resistive strength training, gym activities, walking programs, cycling or aquatic therapy and falls prevention education programs have not been reported in the PSP literature.

There were some limitations of this analysis. Firstly, it was derived from a recent systematic review in which only 11 studies, with only 99 participants in total, met the eligibility criteria. Whether those studies reflect contemporary clinical practice is unknown. For the current analysis, rigorous methods were used to identify research reports and assess the reporting quality in the included studies. A CERT Explanation and Elaboration Statement that provides guidance specifically for complex neurological rehabilitation could be explored and designed with input from a panel of experts.

For future research, it is important to design interventions that have a comprehensive and explicit protocol that can be replicated. Greater use of online supplemental materials now provided by academic and clinically orientated journals is one method of including additional detail. Journal editors and peer reviewers play an integral role in ensuring that exercise, rehabilitation, physical activity and physical therapy research is complete and detailed. Researchers are urged to include qualitative methods in research design for exploration of individual preferences and capabilities that would inform intervention design.

## Conclusion

Although exercises may have the potential to benefit people living with PSP [20], currently there are few studies that adequately report all key elements of exercise therapy design, implementation and assessment. Overall, explicit reporting of exercise interventions for PSP was found to be problematic within the existing movement disorders literature, making interventions difficult to replicate. It is recommended that future trials use methods such as the CERT to provide a comprehensive description of exercise and physical activity interventions. Journal and editorial policies are also recommended to encourage adherence to reporting guidelines and standards.

## Data Availability

Data are available from the first author on request.

## Abbreviations

CERT: Consensus on Exercise Reporting Template
PD: Parkinson’s disease
PRISMA: Preferred Reporting Items for Systematic review and Meta-Analysis
PROSPERO: International prospective register of systematic reviews
PSP: Progressive Supranuclear Palsy

## References

[1] Ali F, Josephs K (2018). The diagnosis of progressive Supranuclear palsy: current opinions and challenges. Expert Rev Neurother.

[2] Stamelou M, Hoeglinger GU (2013). Atypical parkinsonism: an update. Curr Opin Neurol.

[3] Bluett B, Litvan I (2015). Pathophysiology, genetics, clinical features, diagnosis and therapeutic trials in progressive Supranuclear palsy. Expert Opin Orphan Drugs.

[4] Stamelou M, de Silva R, Arias-Carrión O, Boura E, Höllerhage M, Oertel WH (2010). Rational therapeutic approaches to progressive Supranuclear palsy. Brain.

[5] Takigawa H, Kitayama M, Wada-Isoe K, Kowa H, Nakashima K (2016). Prevalence of progressive Supranuclear palsy in Yonago: change throughout a decade. Brain Behav.

[6] Hoglinger GU, Respondek G, Stamelou M, Kurz C, Josephs KA, Lang AE (2017). Clinical diagnosis of progressive Supranuclear palsy: the movement disorder society criteria. Mov Disord.

[7] Lamb R, Rohrer JD, Lees AJ, Morris HR (2016). Progressive Supranuclear palsy and Corticobasal degeneration: pathophysiology and treatment options. Curr Treat Options Neurol.

[8] Glasmacher SA, Leigh PN, Saha RA (2017). Predictors of survival in progressive Supranuclear palsy and multiple system atrophy: a systematic review and meta-analysis. J Neurol Neurosurg Psychiatry.

[9] Morris ME, Menz H, McGinley JL, Watts JJ, Huxman FE, Murphy HT (2015). Randomized controlled trial to reduce falls in people with Parkinson’s disease. Neurorehabil Neural Repair.

[10] Morris ME, Iansek R, Kirkwood B (2009). A randomised controlled trial of movement strategies compared with exercise for people with Parkinson’s disease. Mov Dissord.

[11] Tomlinson CL, Herd CP, Clarke CE, Meek C, Patel S, Stowe R, et al. Physiotherapy for Parkinson’s disease: a comparison of techniques. Cochrane Database Syst Rev. 2014;6:1–107. 10.1002/14651858.CD002815.pub2 PMC7120367 24936965

[12] Chung CL, Thilarajah S, Tan D (2016). Effectiveness of resistance training on muscle strength and physical function in people with Parkinson's disease: a systematic review and meta-analysis. Clin Rehabil.

[13] Shen X, Wong-Yu ISK, Mak MKY (2015). Effects of exercise on falls, balance and gait ability in Parkinson’s disease: a meta-analysis. Neurorehabil Neural Repair.

[14] Lamont R, Morris ME, Woollacott MH, Brauer SG (2012). Community walking in people with Parkinson’s disease. Park Dis.

[15] Schenkman M, Moore CG, Kohrt WM, Hall DA, Delitto A, Comella CL (2018). Effect of high-intensity treadmill exercise on motor symptoms in patients with de novo Parkinson disease: a phase 2 randomized clinical trial. JAMA Neurol.

[16] Ashok C (2013). Physiotherapy management for progressive Supranuclear palsy. Int J Physiother Res.

[17] Intiso D, Bartolo M, Santamato A, Di Rienzo F (2018). The role of rehabilitation in patients with progressive Supranuclear palsy: a narrative review. PMR.

[18] Tilley E, White S, Peters MD, Koblar SA, McLoughlin J (2014). The effectiveness of allied health therapy in the symptomatic management of: a systematic review protocol. JBI Database System Rev Implement Rep.

[19] Tilley E, McLoughlin J, Koblar SA, Doeltgen SH, Stern C, White S (2016). Effectiveness of allied health therapy in the symptomatic management of progressive Supranuclear palsy: a systematic review. JBI Database System Rev Implement Rep.

[20] Slade SC, Finkelstein D, McGinley J, Morris ME. Exercise and physical activity for people with progressive Supranuclear palsy: a rare atypical parkinsonism. Clin Rehabil. 2019. 10.1177/0269215519877235. 10.1177/0269215519877235 PMC6943961 31559853

[21] Zampieri C, di Fabio RP (2008). Balance and eye movement training to improve gait in people with progressive Supranuclear palsy: quasi-randomized clinical trial. Phys Ther.

[22] Zampieri C, Di Fabio RP (2009). Improvement of gaze control after balance and eye movement training in patients with progressive Supranuclear palsy: a quasi-randomized controlled trial. Arch Phys Med Rehabil.

[23] Clerici I, Ferrazzoli D, Maestri R, Bossio F, Zivi I, Canesi M, Frazzitta G (2017). Rehabilitation in progressive Supranuclear palsy: effectiveness of two multidisciplinary treatments. PLoS One.

[24] Nicolai S, Mirelman A, Herman T, Zijlstra A, Mancini M, Becker C (2010). Improvement of balance after audio-biofeedback. A 6-week intervention study in patients with progressive Supranuclear palsy. Z Gerontol Geriatr.

[25] Sale P, Stocchi F, Galafate D, De Pandis MF, Le Pera D, Sova I (2014). Effects of robot assisted gait training in progressive Supranuclear palsy (PSP): a preliminary report. Front Hum Neurosci.

[26] Wittwer JE, Winbolt M, Morris ME (2019). A home-based, music-cued movement program is feasible and may improve gait in progressive Supranuclear palsy. Front Neurol.

[27] Slade SC, Keating JL (2012). Exercise prescription: a case for standardised reporting. Br J Sports Med.

[28] Slade SC, Dionne CE, Underwood M, Buchbinder R (2016). Consensus on exercise reporting template (CERT): a modified Delphi study. Phys Ther.

[29] Slade SC, Dionne CE, Underwood M, Buchbinder R (2016). The consensus on exercise reporting template (CERT): explanation and elaboration statement. Brit J Sports Med.

[30] Kent P, O’Sullivan PB, Keating JL, Slade SC (2018). Evidence-based exercise prescription is facilitated by the consensus on exercise reporting template (CERT). Brit J Sports Med.

[31] Higgins J, Green S. Cochrane handbook for systematic reviews of interventions. Version 5.3.0 (updated October 2015): The Cochrane Collaboration; 2015. https://www.handbook.cochrane.org.

[32] Moher D, Liberati A, Tetzlaff J, Altman DG (2009). The PRISMA group. Preferred reporting items for systematic reviews and meta-analyses: the PRISMA statement. Ann Intern Med.

[33] Liberati A, Altman DG, Tetzlaff J, Mulrow C, Gøtzsche PC, Ioannidis JPA (2009). The PRISMA statement for reporting systematic reviews and meta-analyses of studies that evaluate health care interventions: explanation and elaboration. PLoS Med.

[34] McGregor G, Powell R, Finnegan S, Nichols S, Underwood M (2018). Exercise rehabilitation programmes for pulmonary hypertension: a systematic review of intervention components and reporting quality. BMJ Open Sport Exer Med.

[35] Suteerawattananon M, MacNeill B, Protas EJ (2002). Supported treadmill training for gait and balance in a patient with progressive Supranuclear palsy. Phys Ther.

[36] Irons SL, Brusola GA, Buster TW, Burnfield JM (2015). Novel motor-assisted elliptical training intervention improves 6-minute walk test and oxygen cost for an individual with progressive Supranuclear palsy. Cardiopulmonary Phys Ther J.

[37] Seamon B, DeFranco M, Thigpen M (2017). Use of the Xbox Kinect virtual gaming system to improve gait, postural control and cognitive awareness in an individual with progressive Supranuclear palsy. Disabil Rehabil.

[38] Wallace R, Abbott C, Gibson-Horn C, Skubic M (2013). In-home measurement of the effect of strategically weighted vests on ambulation. 2013 35th Annual International Conference of the IEEE Engineering in Medicine and Biology Society (EMBC).

[39] Di Pancrazio L, Bellomo RG, Franciotti R, Iodice P, Galati V, D’Andreagiovanni A, Saggini R (2013). Combined rehabilitation program for postural instability in progressive supranuclear palsy. Neuro Rehabil.

[40] Williams N (2017). The Borg rating of perceived exertion (RPE) scale. Occup Med.

[41] Maclean N, Pound P (2000). A critical review of the concept of patient motivation in the literature on physical rehabilitation. Soc Sci Med.

[42] McGrane N, Cusack T, O’Donoghue G, Stokes E (2014). Motivational strategies for physiotherapists. Phys Ther Rev.

[43] O’Halloran PD, Blackstock F, Shields N, Holland A, Iles R, Kingsley M (2014). Motivational interviewing to increase physical activity in people with chronic health conditions: a systematic review and meta-analysis. Clin Rehabil.

[44] Volk ML, Lieber SR, Kim SY, Ubel PA, Schneider CE (2011). Contracts with patients in clinical practice. Lancet.

[45] Warburton DER, Bredin SSD (2017). Health benefits of physical activity: a systematic review of current systematic reviews. Curr Opin Cardiol.

[46] Silva NL, Oliveira RB, Fleck SJ, Leon ACMP, Farinatti P (2014). Influence of strength training variables on strength gains in adults over 55 years-old: a meta-analysis of dose response relationships. J Med Sci Sport.

